# Supplementary material for: Culicoides (Diptera: Ceratopogonidae) Abundance Is Influenced by Livestock Host Species and Distance to Hosts at the Micro Landscape Scale
Source: Insects. 2023 Jul 14;14(7):637. doi: 10.3390/insects14070637 (PMC10380773; doi:10.3390/insects14070637)
Supplement: Supplementary file 1 [file insects-14-00637-s001.zip › insects-2463315-supplementary.pdf]

Table S1. Generalized linear mixed (best fit) Poisson model fitted to quantify the mean number of female *Culicoides* sweep-netted in the neighborhood of different baits and by distance to baits.

Variable	Estimate	std error	statistic	p-value	lower 95% confidence limit estimate	higher 95% confidence limit estimate
Intercept (cow)	6.143	0.204	30.044	0.000	5.742	6.544
Sheep	-2.580	0.208	-12.382	0.000	-2.988	-2.171
Light trap	-4.052	0.227	-17.885	0.000	-4.496	-3.608
Natural splines at distance up to 10 m	-4.237	0.384	-11.037	0.000	-4.989	-3.485
Natural splines at distance higher than 10 m	-1.674	0.197	-8.504	0.000	-2.059	-1.288
Interaction between sheep and natural splines at distance up to 10 m	3.915	0.557	7.027	0.000	2.823	5.007
Interaction between light trap and natural splines up to 10 m	6.046	0.590	10.245	0.000	4.890	7.203
Interaction between sheep and natural splines at distance higher than 10 m	1.028	0.284	3.618	0.000	0.471	1.586
Interaction between light trap and natural splines at distance higher than 10 m	2.473	0.285	8.673	0.000	1.914	3.032
Random Effects						
Observational effect (Sd) [1]	0.4926841					
day (Sd)[2]	0.5061843					

[1] daily sources of variation due to influence of weather (temperature and humidity) and daily repeated measures on the same baits

[2] daily varying overdispersion (observation effects) on the *Culicoides* distribution

Table S2. Generalized linear mixed (best fit) Poisson model fitted to quantify the rate of blood-fed female *Culicoides* sweep-netted in the neighborhood of different baits and by distance to baits.

Variable	Estimate	std error	statistic	p-value	lower 95% confidence limit estimate	higher 95% confidence limit estimate
Intercept (cow)	-2.630	0.210	-12.526	0.000	-3.041	-2.218
Sheep	-0.105	0.296	-0.353	0.724	-0.685	0.476
Light trap	0.162	0.327	0.496	0.620	-0.478	0.802
Natural splines at distance up to 10 m	-2.304	0.308	-7.468	0.000	-2.908	-1.699
Natural splines at distance higher than 10 m	-0.640	0.276	-2.318	0.020	-1.181	-0.099
Random Effects						
Day[1]	0.36975878					
Sampler[2]	0.2452485					

[1] daily sources of variation due to influence of weather (temperature and humidity) and daily repeated measures on the same baits

[2] Variation introduced by different people performing the catches
